# *De novo* design of antibody complementarity determining regions binding a FLAG tetra-peptide

**DOI:** 10.1038/s41598-017-10737-9

**Published:** 2017-08-31

**Authors:** Kevin C. Entzminger, Jeong-min Hyun, Robert J. Pantazes, Athena C. Patterson-Orazem, Ahlam N. Qerqez, Zach P. Frye, Randall A. Hughes, Andrew D. Ellington, Raquel L. Lieberman, Costas D. Maranas, Jennifer A. Maynard

**Affiliations:** 1 Department of Molecular Biosciences, University of Texas at Austin, Austin, TX 78712 USA; 2 Department of Chemical Engineering, University of Texas at Austin, Austin, TX 78712 USA; 3 Applied Research Laboratories, University of Texas at Austin, Austin, TX 78712 USA; 4 Department of Chemical Engineering, Auburn University, Auburn, AL 36849 USA; 5 School of Chemistry and Biochemistry, Georgia Institute of Technology, Atlanta, GA 30332 USA; 6 Department of Chemical Engineering, Pennsylvania State University, University Park, Pennsylvania, PA 16802 USA

## Abstract

Computational antibody engineering efforts to date have focused on improving binding affinities or biophysical characteristics. *De novo* design of antibodies binding specific epitopes could greatly accelerate discovery of therapeutics as compared to conventional immunization or synthetic library selection strategies. Here, we employed *de novo* complementarity determining region (CDR) design to engineer targeted antibody–antigen interactions using previously described *in silico* methods. CDRs predicted to bind the minimal FLAG peptide (Asp–Tyr–Lys–Asp) were grafted onto a single-chain variable fragment (scFv) acceptor framework. Fifty scFvs comprised of designed heavy and light or just heavy chain CDRs were synthesized and screened for peptide binding by phage ELISA. Roughly half of the designs resulted in detectable scFv expression. Four antibodies, designed entirely *in silico*, bound the minimal FLAG sequence with high specificity and sensitivity. When reformatted as soluble antigen-binding fragments (Fab), these clones expressed well, were predominantly monomeric and retained peptide specificity. In both formats, the antibodies bind the peptide only when present at the amino-terminus of a carrier protein and even conservative peptide amino acid substitutions resulted in a complete loss of binding. These results support *in silico* CDR design of antibody specificity as an emerging antibody engineering strategy.

## Introduction

Antibodies are one of the most important protein classes, widely used in commercial diagnostics and therapeutics that garnered global sales of $75 billion in 2013^1^. They have conventionally been developed through experimental approaches, such as animal immunization followed by hybridoma generation and, more recently, screening of synthetic libraries. These approaches are limited for targets that harbor poorly accessible epitopes or require precise molecular engagement to achieve the desired biological effects. Moreover, since sequence diversity expands at a rate of 20^n^, where n is the number of randomized amino acids, synthetic library sizes rapidly approach the limit of what can be reasonably screened using display technologies. Computational approaches have the potential to dramatically reduce the resources required for antibody discovery while increasing success rates for challenging targets. The growing utility of *de novo* protein design is demonstrated by a number of recent successes with therapeutic potential, including the design of anti-HIV^2^ and antimicrobial peptides^3^, epitope mimics for vaccination^4^ and influenza inhibitors^5^.

Antibody-antigen interactions are dominated by the complementarity determining regions (CDRs), three on each of the heavy and light variable domains. Typically, CDRs bind antigens by forming a shape-complementary pocket with favorable interactions distributed throughout the CDRs. Although there are exceptions to this typical binding mode, such as the VRC01 class of broadly neutralizing anti-HIV antibodies^6^, they are rare. Soon after the first antibody structures were solved, it was recognized that CDR backbones cluster into distinct groups of canonical structures^7^ with unique amino acid sequence preferences. This observation has facilitated the development of several methods for predicting antibody tertiary structures from their amino acid sequences^8^. However, despite successes in *de novo* protein design in general, computational antibody engineering to date has typically focused on improving characteristics of existing antibodies rather than designing novel specificities. Examples include identifying charged mutations that confer thermo-resistance^9^, guiding affinity maturation^10^, improving association rates^11^ and identifying aggregation prone regions^12^. A recent exception to this trend was the development of a method to rationally design antibodies to bind epitopes in disordered portions of the antigen^13^. While effective, this method uses an atypical binding mode and is limited to epitopes in disordered regions of protein structure.

To address these limitations, a computational method, Optimal CDR (OptCDR) for *de novo* design of antibody binding interfaces complementary to specific three-dimensional epitopes was recently reported^14^. This approach is unique in that it first selects canonical CDR backbone loop structures, then decorates them with specific amino acid side chains, using energy minimization to refine the structure and maximize predicted interactions with the target epitope. Here, we used this approach to design CDRs binding the minimal FLAG peptide (sequence: DYKD)^15^. The FLAG peptide is widely used for protein detection and affinity purification in conjunction with commercially available antibodies binding the extended form of the peptide. Additionally, the FLAG peptide has been incorporated into external protein loops without altering core structure^16^, allowing for its use in combination with an anti-FLAG crystallization chaperone to guide high-throughput structural biology efforts^17^. As a target sequence, its short length restricts the range of possible conformations while its residues are capable of forming nonpolar, hydrogen-bonding and cation-π interactions. Moreover, the FLAG peptide has been observed to form specific structures^18^ and thus is a candidate to assess our ability to design antibodies binding small, conformational epitopes versus more flexible linear epitopes.

Two libraries were created by full design of all six CDRs (EEf library) or just the three heavy chain CDRs (EEh library). Ten EEf and forty EEh designs were synthesized as single chain variable fragments (scFv) and screened by phage ELISA for binding to the FLAG peptide when presented by a carrier protein. Four antibodies, two from each library, exhibited FLAG peptide binding which was maintained after conversion to a soluble antigen binding fragment (Fab) format. These antibodies bind the minimal four residue FLAG peptide with high sensitivity (ELISA half maximal effective concentrations [EC_50_] ranging from 4–50 nM) and are extremely specific, as even conservative substitutions in the peptide sequence result in complete loss of binding. As Fabs, they expressed well, were predominantly monomeric and retained binding after several months of storage. These results support *de novo* CDR design to target specific epitopes as a viable engineering approach.

## Results

### *In silico* antibody design

The OptCDR method *de novo* designs antibody CDRs against any specified antigenic epitope^14^. Briefly, OptCDR starts with a database of canonical structure backbones for each CDR derived from known antibody structures. This includes CDR H3, which is not considered to have canonical structures in the antibody literature and for which OptCDR has ten-fold more structures than any other CDR. By including a diverse but finite number of CDR H3 structures, OptCDR treats all six CDRs equivalently. Given a position of the antigen, for each canonical structure, OptCDR calculates a geometric score where CDR backbone atoms close to the antigen are rewarded as having the potential to contribute to binding but van der Waals clashes are penalized. A mixed-integer linear programming (MILP) optimization formulation then selects the combination of canonical structures with the best score. Generating thousands of random antigen positions leads to the identification of high-scoring antigen position/canonical structure combinations. Because the canonical structures only contain backbone atoms, the rotamer library and rotamer-selection MILP from the IPRO Suite^19^ are used to fill in the amino acid side chains of the canonical structures while obeying both CDR and canonical structure specific sequence rules. Finally, several thousand iterations of the IPRO algorithm are used to computationally affinity mature the CDRs through the identification of favorable mutations.

As the framework for the designed CDRs, an scFv with known structure was selected that we previously engineered to bind an EE peptide (αEE scFv; EE sequence EYMPME)^20^. The carrier protein used in binding assays was previously crystallized with an n-terminal DYKD tag (PDB: 3ESU, 3ESV, 3ET9, 3ETB)^18^. Only one of the four structures (3ESV) has three of the four tag residues resolved, although the D in peptide position P4 is resolved in all structures. In 3ESV, the kinked conformation of DYKD is stabilized by a hydrogen bond between this D in peptide position P1 and a Q on the carrier protein. Together, these observations suggest that the structure of DYKD in this system is moderately mobile with some stabilization provided by the carrier protein. Since a full structure of the target was not known, a model of the FLAG peptide was built as described in the methods. The minimal DYKD sequence was specified as the epitope in the design calculations. While an intact antibody has six CDRs, the three heavy chain CDRs can be sufficient to confer novel specificity^21^. Therefore, two libraries were designed: EEf, in which all six CDRs were designed to fully test the modeling process, and EEh, in which only the three heavy chain CDRs were designed and the light chain was not modified.

For each library, 30 preliminary designs were generated. The top 15 designs, composed of three EEf library (Table S1) and twelve EEh library (Table S2) members and determined by calculated interaction energies, were then affinity-matured computationally. The computational interaction energy, defined as the minimized energy of the CDR-antigen complex minus the energies of the CDRs and antigen individually, ranged from −191 to −609 kcal/mol for EEf designs (Table S3) and from −129 to −476 kcal/mol for EEh designs (Table S4). More negative energies indicate favorable interactions, but the interaction energies serve as a guide and are not expected to be quantitatively predictive of experimental binding affinities. Contacts are defined as the number of CDR atoms (carbon, nitrogen and oxygen) within 3 Å of the antigen and respectively ranged from 52–78 and 21–60 for the EEf and EEh designs. Polar contacts were determined using the PyMOL Molecular Graphics System (PyMOL) and ranged from 8–12 and 1–10 for the EEf and EEh designs, respectively. Across all three computational metrics, there was a trend of superior values for the EEf designs. This is a result of constraining the EEh library light chain to its wild-type structure, preventing its optimization *in silico*.

For each selected antibody, several derivative designs were also chosen for experimental characterization. These designs correspond to intermediate CDR amino acid sequences identified during the computational affinity maturation, but prior to the final design being selected. Designs were selected such that they collectively distributed differences throughout as many CDRs as possible. Antibodies are labeled according to whether all six or just the three heavy chain CDRs were designed (EEf or EEh, respectively), followed by a preliminary design number (1–30) and then a derivative design number (0.0–0.6), in which increasing values correspond to increasing numbers of residue changes relative to the preliminary design suggested by computational affinity maturation. Despite better computational predictions, ultimately more EEh than EEf antibodies were selected for experimental characterization based on the hypothesis that grafting fewer CDRs would be less likely to disrupt the framework scFv and thus more likely to yield well-behaved proteins. In total, 10 EEf and 40 EEh designs were synthesized as scFv genes.

### Identification of antibodies with peptide-binding activity

**Figure 1:**
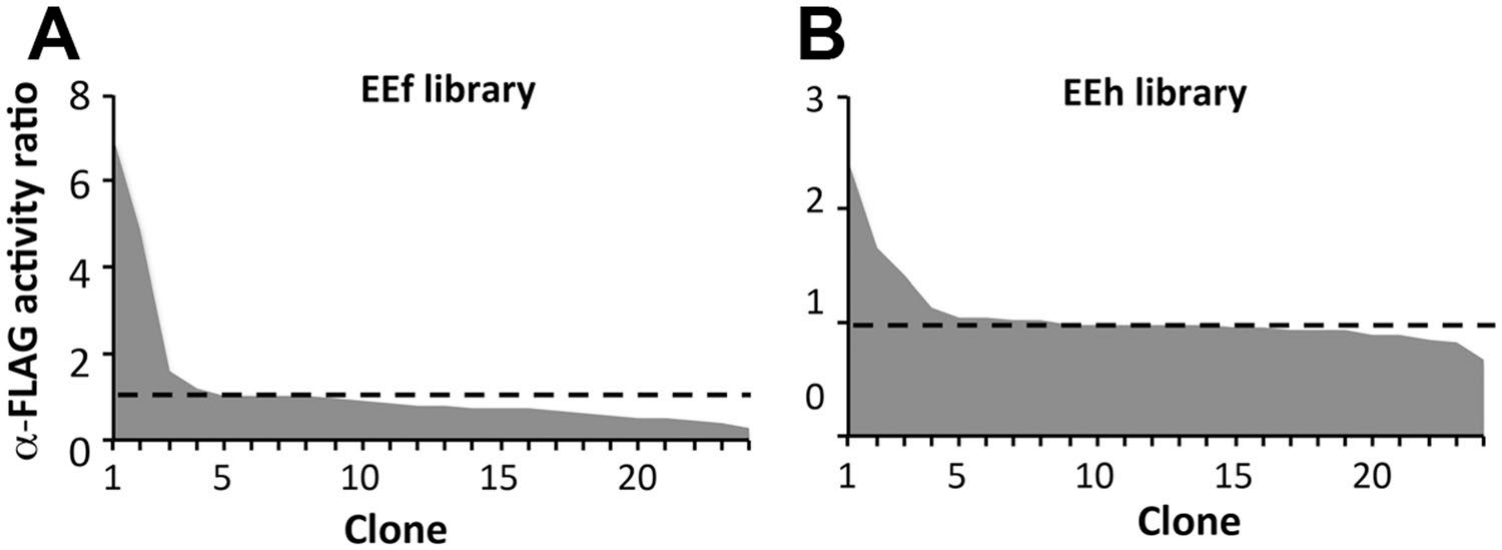
Multiple OptCDR designs exhibit peptide-binding activity as scFv-phage. (**A**) Screening of the EEf and (**B**) EEh libraries. The library gene synthesis products for each library were pooled and cloned *en masse* into a phagemid. The resulting scFv-bearing phage were produced from single colonies and assessed for scFv expression level and peptide-binding activity by ELISA. Shown is the specific activity for each clone, determined as the ratio of the absorbance at 450 nm for an n-DYKD well over that for well coated with carrier protein lacking any FLAG peptide. The dashed line indicates the average non-specific binding signal for an unrelated control scFv.

To test the behavior of the selected designs, scFv genes in the V_L_-linker-V_H_ orientation were constructed using automated protein fabrication. The synthesis products for each individual library were pooled, cloned *en masse* into a phagemid display vector and transformed into *Escherichia coli*. Cloning errors were determined to be less than 5% based on colony PCR, while gene synthesis errors were found to be present in nearly 60% of clones by DNA sequencing, primarily localized to the repetitive GlySer linker region. Accounting for synthesis and cloning errors, sufficient colonies were screened to sample the library size at about three times coverage (72 clones for EEf and 368 for EEh). Phage from individual colonies were propagated and tested by ELISA for binding to three forms of the FLAG peptide: the minimal DYKD tag at the n-terminus and the full-length DYKDDDDK peptide at the n- or c-terminus of the same carrier protein (Figure S1). The peptide binding activity, defined as the raw absorbance value from a peptide-coated well divided by the raw absorbance value from a control well coated with naked carrier protein, is reported for each clone. The EEf library included multiple clones binding the DYKD-protein with activity ratios up to seven, while the EEh library includes clones with ratios up to 2.5 (Fig. 1). Much weaker binding was observed for the highly-charged, extended FLAG epitope of DYKDDDDK in either the n- or c-terminal position (data not shown). The range of scFv display levels was similar for both libraries, as assessed by binding of a c-myc peptide tag at the c-terminus of the scFv to an anti-c-myc antibody by ELISA. One-third (EEf) to one-half (EEh) of clones from each library exhibited anti-c-myc strong signal, indicating that display level likely did not contribute to observed differences in library performance (Figure S2).

**Figure 2:**
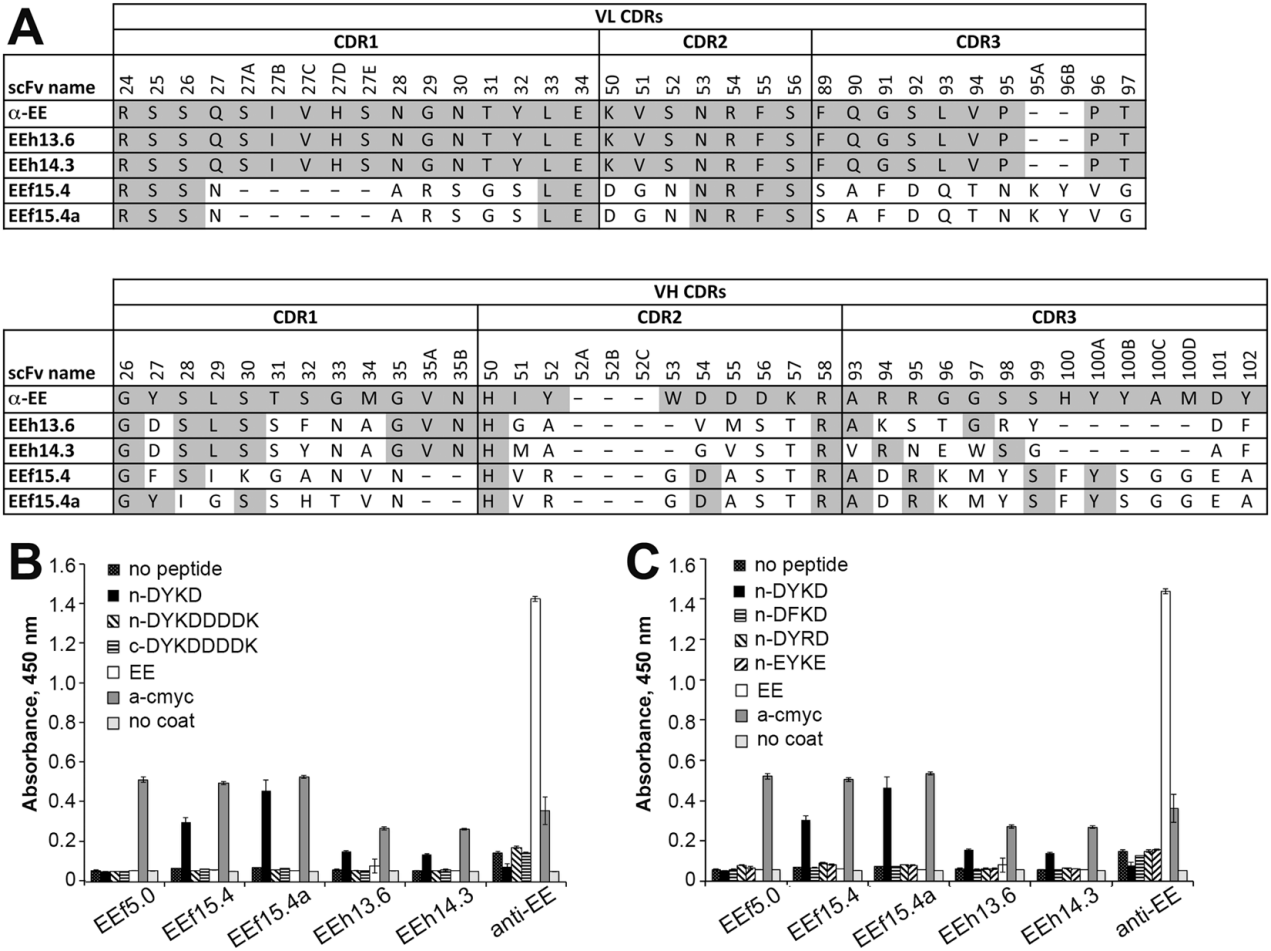
Designed antibodies bind DYKD peptide as scFv-phage. (**A**) Alignment of CDR sequences for clones selected from the initial screen. The αEE scFv used as the acceptor framework to present the designed CDRs is shown as a reference. Clones EEh13.6 and EEh14.3 were selected from the EEh library and thus share light chain CDRs with αEE. Clones EEf15.4 and EEf15.4a were selected from the EEf library and differ only in CDR H1. No amino acid differences were observed outside the CDRs. Kabat numbering is indicated, with CDRs defined according to OptCDR’s sequence-based rules. (**B**) To determine the effect of peptide location, binding of scFv-displaying phage was assessed by monoclonal phage ELISA to the carrier protein with no tags, or with an n-terminal DYKD tag, n-terminal DYKDDDDK tag or c-terminal DYKDDDDK tag. (**C**) To assess peptide specificity, monoclonal phage ELISA was used to determine binding to peptide variants including n-DFKD, n-DYRD and n-EYKE. For both ELISAs, controls included the anti-c-myc antibody to assess scFv expression levels and naked carrier protein, EE peptide and uncoated wells to assess non-specific binding. Purified phage were titrated to collect a full dose-response curve; shown is the raw absorbance at 450 nm for wells containing 10^11^ pfu, which was either in the linear dose-response or background range for each curve. The mean and standard deviation are shown; each experiment was repeated at least twice with independent phage preparations.

Clones from each library with high activities were sequenced to reveal four unique sequences with varying CDR lengths and sequences: EEh13.6, EEh14.3, EEf15.4 and EEf15.4a, the latter two differing only in CDR H1 (Fig. 2A). Clone EEf15.4a is the result of a gene synthesis error that combined the H1 CDR from EEf5.1 with the other five CDRs of EEf15.4. Designs EEf15 and EEf5 had the same selected canonical structure for the H1 CDR with similar positions of DYKD in their binding pockets, so it is not surprising that this change was tolerated. Thus, EEf15.4a is composed of six CDRs that were entirely computationally designed.

To confirm peptide binding activity and specificity, monoclonal phage ELISAs were performed with the four clones and the parental αEE scaffold antibody. Phage were titrated on wells coated with a carrier protein presenting the minimal DYKD peptide at the n-terminus, the full-length DYKDDDDK peptide at the n- or c-termini, or an EE-peptide (sequence: EYMPME; Figure S3). Control ligands included a tag-free carrier protein, an anti-c-myc antibody to monitor scFv display level and uncoated wells to monitor non-specific binding, respectively. Each of the four unique clones identified in the screen bound only to the minimal DYKD peptide in the n-terminal position, while the parental αEE protein used for the framework bound only to the EE peptide (Fig. 2B, Figure S4A). A control library clone (EEf5.0) that did not exhibit a high activity ratio in the initial screen but exhibited a high level of scFv display showed no binding to any peptide ligand.

To assess the role of each peptide residue in antibody recognition, a series of conservative changes were introduced into the peptide at the n-terminus of the carrier protein: DFKD, DYRD and EYKE. Remarkably, antibody binding to each of these variants was similar to background binding levels (Fig. 2C, Figure S4B). These data demonstrate that these *de novo* designed antibodies bind only the minimal FLAG peptide when present at the n-terminus and that each residue in the minimal DYKD peptide is essential for binding.

### Designed antibodies produce high quality protein as soluble Fab

**Figure 3:**
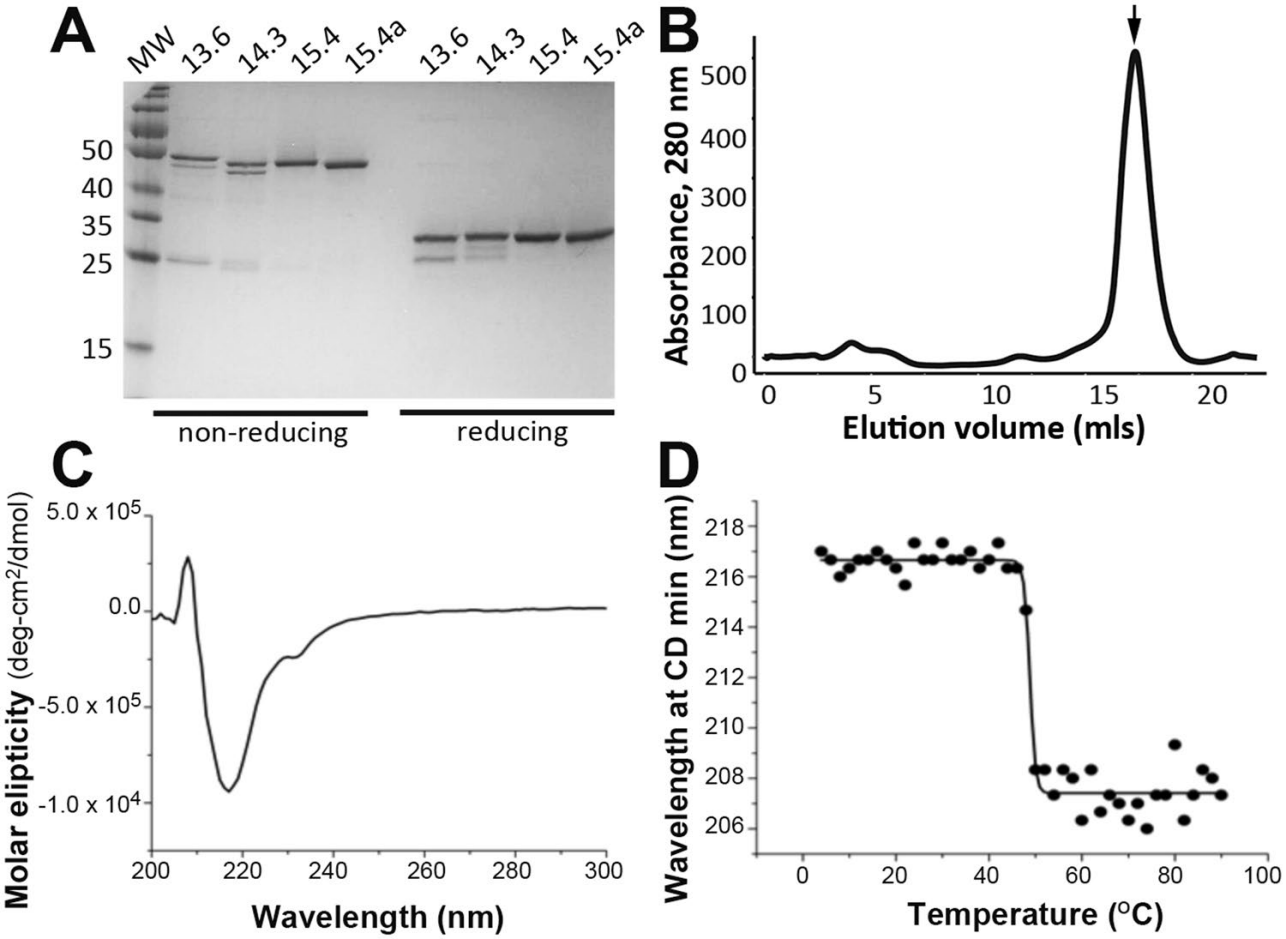
*De novo* designed Fab antibodies yield well-expressed, folded protein. Fabs were expressed as soluble periplasmic proteins in 250 ml bacterial cultures, purified by osmotic shock followed by immobilized metal affinity chromatography and size exclusion chromatography. (**A**) Purified Fabs were assessed for purity and size by SDS-PAGE. Shown are reduced and non-reduced samples loaded with 2 μg protein per lane with a blank lane in between the non-reduced and reduced samples. This image is of a single gel which has not been modified to enhance contrast or exposure. (**B**) The preparative size exclusion chromatographic trace on a Superdex S200 column is shown for Fab EEf15.4. The monomeric Fab peak is indicated with an arrow. (**C**) Representative CD spectrum of monomeric Fab EEf15.4 acquired at 4 °C demonstrates the expected high β-sheet content for an Fab. (**D**) Thermal melt data for Fab EEf15.4. The midpoint of the melting curve is 49 ± 1 °C, calculated as the average and standard deviation over two independent protein samples.

To determine whether these antibodies retain DYKD peptide binding activity as purified proteins, the variable regions were cloned into a bacterial periplasmic Fab expression vector, expressed and purified by sequential metal affinity and size exclusion chromatographic steps. Fab EEf15.4 and EEf15.4a expressed at ~10 mg/L culture as predominantly monomeric protein, while Fabs EEh14.3 and EEh13.6 had reduced yield (~2 mg/L and 0.6 mg/L, respectively; Table 1) and the latter co-purified with free light chain. SDS-PAGE showed all Fabs migrated at ~50 kDa under reducing conditions and as a doublet of ~25 kDa under non-reducing conditions (Fig. 3A). Preparative size exclusion chromatography showed a high yield of predominantly monomeric protein (Fig. 3B, Figure S5). Circular dichroism (CD) spectra of Fab EEf15.4 indicated the presence of a typical β-sheet signature with a minimum at 217 nm and a second feature at 230 nm (Fig. 3C). The midpoint of thermal unfolding for Fab EEf15.4 was 49 ± 1 °C, as measured by CD (Fig. 3D). This is lower than the previously measured value of 59.8 ± 0.1 °C for the parental αEE Fab^22^, indicating that the stability of EEf15.4 is lower than that of a typical antibody generated by *in vivo* somatic hypermutation. Regardless, analytical size exclusion chromatography of the purified Fab peak performed after two months of storage at 4 °C showed that the proteins remained predominantly monomeric, indicting favorable storage stability (Figure S6).

**Table 1:** Biochemical properties of *de novo* designed FLAG-binding Fabs.

	Expression level (mg/L culture)	% Monomer	Melting temperature (°C)	ELISA EC_50_ (nM)*
EEh13.6	0.4 ± 0.1	33	nd	21.0 ± 2.7
EEh14.3	2.0 ± 0.3	79	nd	50.0 ± 1.5
EEf15.4	9.4 ± 1.0	95	49 ± 1	4.1 ± 0.9
EEf15.4a	10.0 ± 0.3	95	nd	4.8 ± 1.1
αEE	2.2 ± 0.5	87	59.8 ± 0.1	35.3 ± 9.3

*ELISA EC_50_ values measured for binding to the n-DYKD ligand, except αEE was measured for binding the EE peptide. All data represents the average and standard deviation of triplicate experiments with multiple protein preparations.

### Designed antibodies retain peptide specificity as soluble Fab

**Figure 4:**
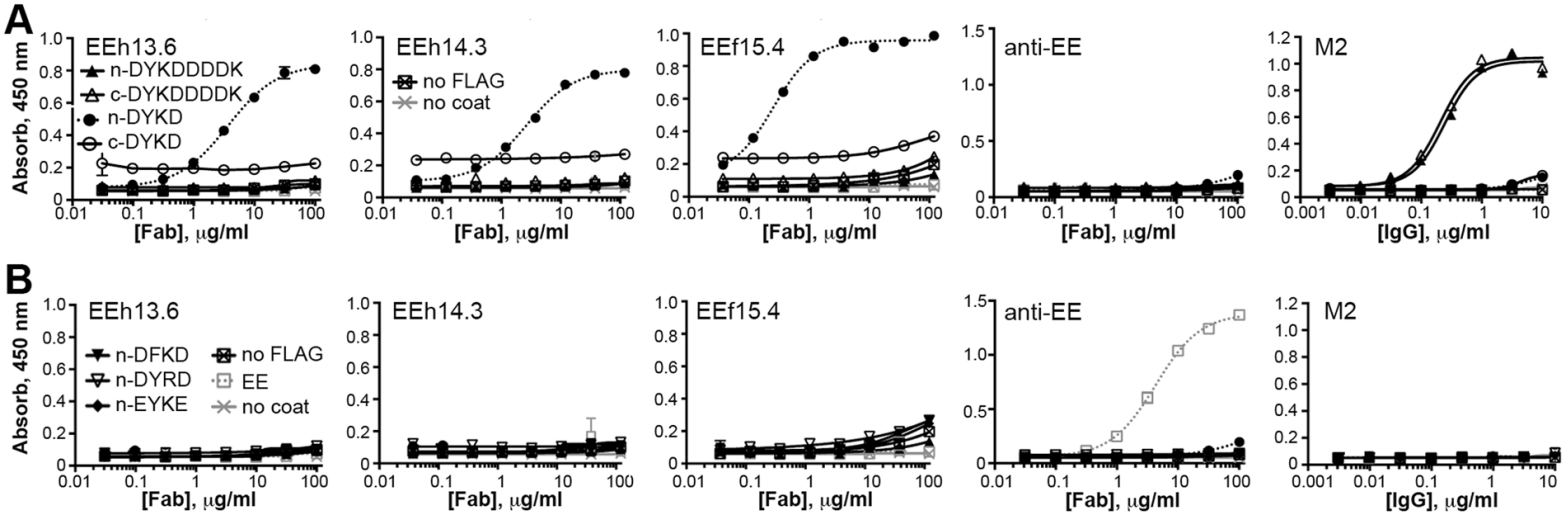
Designed Fab proteins specifically bind the DYKD peptide. The purified Fab proteins were assessed for binding to different FLAG and control peptides presented by the same carrier protein. (**A**) The selected Fabs bind to the DYKD peptide only when presented at the n-terminus. Ligands tested include the minimal DYKD and full-length FLAG peptides presented at the n- or c-terminus or the carrier protein. (**B**) The selected Fabs specifically bind the DYKD sequence; even conservative amino acid substitutions obliterate binding. The n-DYKD ligand was altered to present the conservative substitutions DFKD, DYRD or EYKE and used in ELISAs. As controls, the αEE Fab binds only the EE peptide while the commercial M2 IgG antibody binds the full-length FLAG peptide when present at the n- or c-terminus but not the minimal DYKD peptide. The experiment was repeated at least twice with different protein preparations; shown is the average and range of the recorded signal; EC_50_ values in nanomolar units (1 μg/ml Fab = 20 nM) are reported in Table 1.

To evaluate the peptide binding specificity of soluble Fab proteins, we repeated ELISAs with the same peptide variants and control proteins. As with the phage-displayed scFvs, strong binding to the minimal DYKD peptide was observed only when present at the n-terminus, with no binding observed for the full-length FLAG peptide, the EE peptide or a control ligand lacking any peptide (Fig. 4A, Figure S7). The Fab concentrations resulting in half maximal ELISA signals (EC_50_) ranged from 4–50 nM (Table 1), indicating the Fabs bind peptide tightly. As was observed with the scFv phage binding, any change to the DYKD peptide, even a single conservative tyrosine to phenylalanine substitution resulted in complete loss of binding (Fig. 4B). The αEE Fab which provided the framework for the OptCDR designs bound its cognate EE peptide with a similar high sensitivity, but did not bind any FLAG ligand.

Several commercial anti-FLAG peptide antibodies are available, which exhibit varying peptide specificities: M1 binds the peptide only when presented at the extreme n-terminus of a protein and requires calcium ions, M5 binds the peptide only when fused to the n-terminus and preceded by a methionine, while the M2 antibody binds the full-length peptide at any terminal or internal position. In this work, the M2 antibody bound the full-length FLAG peptide when presented at the n- or c-terminus of our carrier protein but did not bind the n-terminal DYKD construct recognized by our designed antibodies (Fig. 4B). Taken together, these data provide precedence for the strict peptide positional requirements for antibody binding observed with our OptCDR designed antibodies. They also suggest that our designed antibodies do not simply recapitulate the binding mechanisms used by previously described anti-FLAG antibodies.

### Predicted antibody-peptide interactions

**Figure 5:**
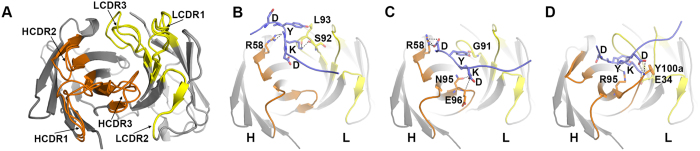
OptCDR predictions of antibody-peptide binding modes. The following coloring was used in each panel of this figure: the modeled peptide is in blue with the DYKD residues shown as stick structures, the heavy chain framework is dark gray with the heavy chain CDRs in orange, while the light chain framework is in light gray with the light chain CDRs in yellow. (**A**) An overlay of the three predicted antibody structures, EEh13.6, EEh14.3 and EEf15.4, with each CDR position highlighted. The CDR residue alignments are shown in Fig. 2A. (**B**–**D**) Show the key predicted interactions between DYKD and EEh13.6, EEh14.3 and EEf15.4, respectively, with each antibody in the same orientation as in panel (A). Each interaction incudes several predicted hydrogen bonds, shown as dashed black lines. In EEh13.6, the backbones of light chain residues S92 and L93 and heavy chain residue R58 are positioned to form hydrogen bonds with the P3 lysine and the backbone of the P2 tyrosine, respectively. In EEh14.3, all four peptide residues are predicted to form hydrogen bonds with antibody residues: heavy chain residue R58 interacts with the first peptide aspartic acid, the backbone of light chain residue G91 with the peptide tyrosine, the sidechain and backbone of heavy chain residues N95 and E96, respectively, with the peptide lysine and light chain residue Y32 with the backbone of the final peptide aspartic acid. In EEf15.4 and EEf154.a, heavy chain CDR3 residue R95 is positioned to hydrogen bond with the backbone of the peptide P2 tyrosine, and Y100a to form a hydrogen bond with the peptide P4 aspartic acid while CDR L1 residue E34 is predicted to form a hydrogen bond with the P3 lysine. The corresponding PDB files are available as supplementary datasets.

A mechanistic understanding of the antibody–DYKD interactions was developed from the initial OptCDR designs of the antibody-antigen complexes and our experimental data. Each designed antibody was initially predicted to have a similar overall structure, with some variation in the CDRs (Fig. 5A), and to bind the DYKD peptide in a distinct orientation relative to the heavy-light chain interface (Fig. 5B–D). Each antibody was also predicted to form multiple favorable interactions with the antigen, distributed among different CDRs and on both the heavy and light chains. For EEh13.6 and 14.3, DYKD is expected to lie in a shallow groove on the antibody surface with the peptide P2 tyrosine and P3 lysine oriented towards the light chain while the P1 aspartic acid residues interact with the heavy chain (Fig. 5B,C). The Fab EEf15.4 interaction is characterized by a deep hole formed at the heavy-light chain interface in which the peptide P2 tyrosine and P3 lysine are deeply buried. EEf15.4a and EEf15.4 are expected to have similar peptide binding interactions, as they differ only by CDR H1, which is on the periphery of the binding pocket and is not expected to contribute significantly to binding. This is consistent with the observation that these two antibodies performed very similarly in the experimental binding assays.

**Figure 6:**
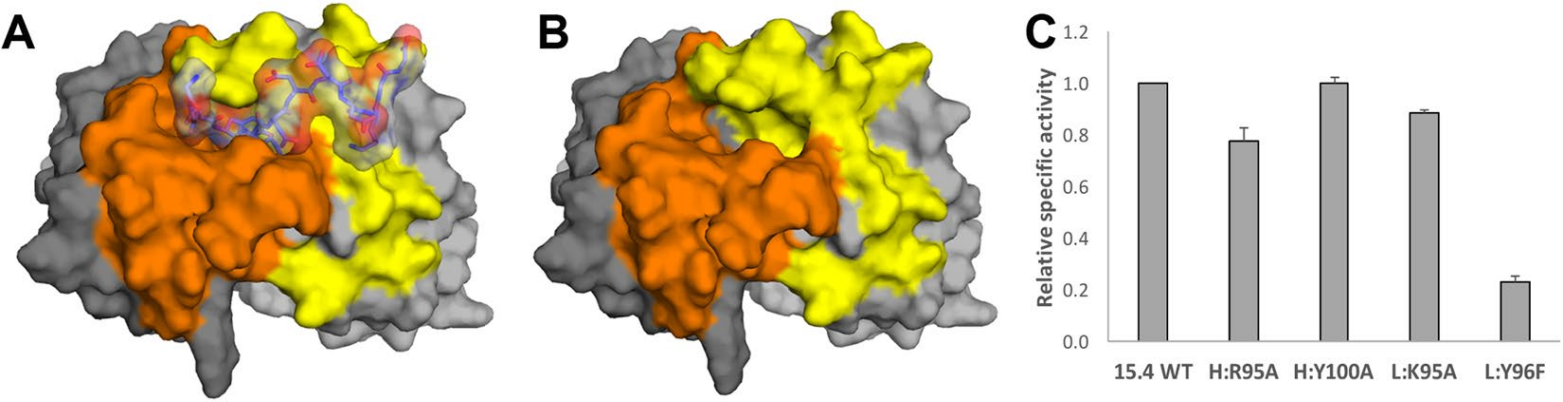
Proposed binding interactions between EEf15.4 and DYKD. The same coloring scheme and antibody orientation was used as in Fig. 5. The EEf15.4 antibody is shown (**A**) with the designed peptide interaction and (**B**) without the peptide. The DYKD residues of the peptide fit into a deep cavity formed by the interface of the heavy and light chain CDRs. The remaining FLAG peptide residues extend over the light chain CDRs but are not predicted to form any interactions. (**C**) Specific binding activity of scFv phage with single amino acid substitutions in CDR residues predicted to be involved in ligand binding. All specific binding values were calculated as the ligand binding signal divided by the anti-c-myc binding signal at a single phage concentration in the linear dose-response regime. These values were then normalized to the specific binding of the unmodified, wild-type EEf15.4 scFv phage (15.4 WT).

It is particularly striking that the EEh antibodies include predicted interactions between the light chain and peptide residues, as modifications to the light chain were prohibited during their design. Those designs may have been successful because the light chain came from a peptide-binding antibody and is therefore predisposed to form a groove compatible with peptide binding. This would then allow OptCDR to place the peptide in such a way that the light chains contribute meaningful binding interactions. The differences between antibodies EEh13.6 and EEh14.3 that exhibited experimental peptide binding and their parental computational designs (i.e. EEh13.0 and EEh14.0, respectively) are on the edges of the binding pockets and are not expected to contribute significantly to binding (Figs 6A and 5B). However, the differences between EEf15.4 and its parental design EEf15.0 are located in CDR H3 and include several amino acids that are predicted to have important interactions with DYKD, suggesting that computational affinity maturation may have improved this interaction.

Looking more closely at EEf15.4, its cavity is predicted to conform to the DYKD structure with an overall shape complementarity value of 0.585^23^. For comparison, a recent survey of antibody-peptide complexes found these to have average shape complementar﻿ity values of 0.75 ± 0.06, while antibody protein complexes were 0.7 ± 0.06^24^. In EEf15.4, the shape complementarity derives from the peptide P2 tyrosine and P3 lysine residues penetrating deeply into the cavity at the antibody heavy-light chain interface, with the P1 and P4 aspartic acid residues sitting near the antibody surface (Fig. 6A,B). The bottom of the pit includes L:Y96 and H:Y100a which may stabilize the P2 tyrosine and P3 lysine through π- and hydrogen-bonding interactions. Along the cavity walls are several charged residues, including H:R95, L:E34 and L:K95 which may interact with the charged peptide residues (Fig. 5D). To provide experimental support for this proposed binding mode, we selected four residues for substitution to remove the indicated interaction (H:R95A, H:Y100A, L:K95A, L:Y96F), followed by scFv phage ELISA to determine the variants’ specific ligand binding activity. After normalizing for scFv protein expression level, H:R95A showed modestly reduced binding (~80%) while L:Y96F showed greatly reduced binding (~20%) relative to the wild-type EEf15.4 (Fig. 6C). These data support a binding mechanism that is similar to that predicted during the design process.

These *de novo* designed antibodies appear to bind ligand in a similar manner as other peptide-binding antibodies. The parent 3D5 antibody, which was used in design of the αEE framework for the OptCDR designs in this study, has been crystallized in complex with its hexa-histidine ligand, revealing that the peptide also binds a groove formed by the antibody heavy and light chain interface^25^. Similarly, the structure of the αEE antibody has a tri-lobed hydrophobic pocket compatible with EYMPME peptide binding^20^. The αEE H:R95 residue was predicted to form polar interactions with multiple peptide side chains, similar to the role it may play in EEf15.4. Interestingly, the predicted interactions of DYKD with the designed antibodies presented here share similarities with the predicted binding mode of the FLAG peptide with the commercial M2 antibody Fab, whose structure was solved^16^. M2 is also predicted to interact primarily with DYKD, which lies on the antibody surface near the heavy-light chain interface. The lysine within DKYD is predicted to form a salt bridge with a glutamic acid residue at the base of an acidic hollow; adjacent to this are two conserved tyrosines that may form π-stacking interactions with the tyrosine in DYKD. Surrounding the tyrosines are two clusters of positively charged residues, which may stabilize the negatively charged aspartic acid residues. However, the successful designs here and M2 share only an “NRFS” motif (residues 53–56) in CDR L2. Another key difference is that M2 can bind the full FLAG peptide at any location within a protein, but not the n-terminal core peptide DYKD recognized by the OptCDR designs.

Finally, each of the successful designs interacts with the peptide in a manner that does not disrupt the DYKD peptide structure observed in PDB 3ESV. In particular, the stabilizing hydrogen bond between the peptide P4 aspartic acid and the carrier protein is maintained and the antibodies interact with FLAG from a direction that is not expected to cause irreconcilable steric clashes with the carrier protein. As discussed in the methods, these features were not constraints used during the design process. Moreover, while all four Fabs bind strongly in ELISA, none bind denatured n-DYKD-carrier protein on a Western blot. Conversely, the commercial M1 antibody binds the same n-DYKD ligand in Western blot but not in ELISA, suggesting the peptide is not fully accessible when fused to the carrier protein (data not shown). These data are consistent with the designed antibodies binding a specific three-dimensional conformation of DYKD, as opposed to the extended linear peptide.

## Discussion

We have performed one of the first successful demonstrations of epitope-specific antibody binding based on *de novo* design. Using two different library approaches, we recovered four unique antibodies with different CDR sequences that each bind the same DYKD peptide conformation, with exquisite specificity. While novel antibody specificities have been rationally designed before^16, 26^, these earlier examples introduced specificity by designing only the H3 CDR via introduction of hydrophobic or β-strand features that target linear epitopes. Although effective, this mode of binding is rare for natural antibodies. Additionally, the only antibodies designed with such methods have been single-domain antibodies (i.e. a V_H_ domain without a V_L_ domain). In contrast, we designed all or several CDRs to bind structural or linear epitopes by forming a pocket that complements the antigen shape with favorable interactions distributed throughout the CDRs and typical CDR sequences. Antibodies binding a dodecapeptide mimic of mannose containing carbohydrates were designed by a similar approach but required several rounds of molecular dynamics optimization and extensive protein refolding efforts^27^, whereas the DYKD-peptide binding antibodies reported here relied solely on the initial computational designs and resulted in high yields of folded protein.

These OptCDR predictions have a remarkably high success rate and notable efficacy. Although the rate of *de novo* protein design successes is increasing, it is still common for tens or hundreds of designs to be screened before one shows the desired activity^28^. At a structural level, it is surprising that three of fifteen unique canonical structure combinations resulted in antigen-binding variants. For antibodies to be viable experimental or therapeutic agents, they must possess good affinities and high specificities, and these antibodies meet those criteria. The four successful designs described here exhibit sensitive binding, which was readily detectable at concentrations of 4–50 nM in ELISA using purified Fab proteins and phage-displayed scFv antibody formats. This result contrasts with common results from *de novo* designed binding interfaces, which usually exhibit micromolar binding affinities^5^ and require experimental affinity maturation to achieve nanomolar binding affinities^29^. Beyond sensitive binding, these four designs are also highly specific for the cognate DYKD peptide, as even conservative changes to this sequence abrogated binding (Figs 3 and 4). While neither OptCDR predictions nor predicted binding modes for homology models are expected to reach atomic level precision, the interactions between DYKD and antibody could be rationalized; future efforts in co-crystallization will provide additional insight.

Moreover, the DYKD peptide appears to bind a specific kinked conformation to the designed antibodies. Since antibody binding requires the peptide be placed at the n-terminus of the carrier protein, interactions with the carrier protein presumably influence the peptide conformation. This binding mode appears possible, as all successful OptCDR designs interact with DYKD such that the antibody residues do not clash with the carrier protein or disrupt peptide-carrier protein interactions. This interpretation also explains why the designed antibodies successfully bound only an n-terminal DYKD and did not bind the linear protein on a Western blot. In contrast, the commercial M1 antibody, which is known to bind a linear FLAG peptide during Western blotting, exhibited the opposite pattern. The shape complementarity metric for the peptide-EEf15.4 antibody interaction was lower than is typically seen for peptide-antibody complexes (0.585 versus 0.75). Since shape complementarity correlated with affinity better than any other biophysical parameter in a previous analysis of lysozyme binding Fabs^30^, this may be a useful metric to prioritize epitope-specific antibody designs in the future.

Although EEh13.6, EEh14.3 EEf15.4 and EEf15.4a bind DYKD specifically and with favorable affinities, our design process did not explicitly address protein folding or stability issues. First, in the EEh library, only four of the ten clones exhibited similar high levels of expression, two of which bound DYKD with high specificity. Second, grafting CDRs into frameworks can be structurally disruptive and/or require compensating changes to framework residues^31^. Notably, PIGS^26^ and RosettaAntibody^32^ predicted main chain breaks in select OptCDR designs (data not shown), suggesting the structures may be poorly matched to the ﻿underlying﻿ framework. This interpretation is supported by the reduced thermal stability of Fab EEF15.4 as compared to the parent αEE Fab (Fig. 5). Together, these data suggest that future OptCDR designs can be improved by screening CDR sequences for (i) reduced aggregation potential, (ii) compatibility with the acceptor framework and (iii) impact on the V_L_-V_H_ interface packing angle, a factor known to be important to proper CDR positioning^10, 33^. To address the latter two concerns, we recently developed a database of modular antibody parts^34^ and an alternative design algorithm, OptMAVEn^35^ for *de novo* design of complete light and heavy variable domains.

Antibodies are the leading class of therapeutics, due in large part to their ability to interact with distinct ligand conformations in order to elicit particular biological responses. However, discovery of new molecules binding specific epitopes is time consuming and challenging, especially for situations requiring a specific antibody-ligand orientation to induce agonist or antagonist effects^36^. We have addressed this challenge by engineering CDRs *de novo* to form a shape-complementary pocket around any specified epitope, here, a tetra-peptide in a specific kinked conformation. The resulting antibodies showed a high rate of specific binding to the targeted DYKD tetra-peptide, thereby demonstrating the viability of epitope-directed antibody engineering by *de novo* CDR design.

## Methods

### Computational antibody design

A model for the complete FLAG peptide (amino acid sequence: DYKDDDDK) ligand was created using the build function in PyMOL and docked to the Fab structure of a partially refined commercial FLAG peptide-binding antibody^16^ using the ClusPro docking server^37^ on antibody mode to orient the peptide in a position likely to be compatible with binding. Two glycine residues were then appended at both ends to mask terminal carboxyl and amine groups. This antigen model was used for OptCDR-guided design with DYKD specified as the epitope as previously described^19^. The backbone conformation of YKD in the model closely matches that in PDB 3ESV. Two competing hypotheses were considered for the performance of the antibodies: that they would recognize only the conformation of the peptide they were modeled to bind or that they would stabilize a peptide that could freely move into the favored conformation. By matching key residues to the conformation in the known structure, the model bridged both hypotheses.

An scFv we previously engineered to bind an EE peptide served as a framework upon which the CDRs were built, as this framework is predisposed to support peptide binding (αEE scFv; EE sequence EYMPME; PDB ID 3NN8)^20^. The framework residues were included in the OptCDR calculations but not permitted to change. Separate approaches were used to create two unique libraries: a full design in which all six CDRs were optimized (termed EEf) and a heavy chain-only design (EEh) that constrained light chain CDR sequences as wild-type. OptCDR design was performed as previously described^14^ to (i) select CDR canonical structure backbones most likely to allow favorable interactions with the antigen, (ii) individually initialize amino acid side chains onto the canonical structure using a rotamer library, energy functions, a mixed-integer linear programming optimization formulation and sequence-based constraints; and (iii) several thousand iterations of the Iterative Protein Redesign & Optimization (IPRO) procedure^19^, which simultaneously refines the CDR backbone structures and amino acid sequences.

### Library synthesis

Genes for all 50 designs were synthesized by protein fabrication automation as described previously^38^. Briefly, amino acid sequences for V_L_ and V_H_ were reverse-translated using an *E. coli* class II codon table and combined in the scFv format in the orientation V_L_-(GGGGS)_4_-V_H_ flanked by bidirectional *Sfi*I cloning sites. The antibody sequences were clustered based on amino acid sequence similarity prior to reverse-translation of the sequences to enable efficient gene synthesis. Sequence sub-groups were then sequentially assembled to make each of the individual antibody genes. The synthesized products for each library were pooled and cloned into the phage display vector pMopac24, which expresses an scFv fused to a truncated *C*-terminal pIII protein and co-expresses the protein chaperone *Skp* to enhance scFv folding. Colony PCR confirmed >95% cloning efficiency, and ten colonies for each library were sequenced to identify the proportion of correctly assembled genes. All oligonucleotides were purchased from Integrated DNA Technologies and all DNA sequencing was performed at the University of Texas at Austin Core Facility.

### Construction of peptide ligand variants

All peptide variants were constructed by site-directed mutagenesis of the pAK400/14B7 plasmid that contains a minimal DYKD epitope at the protein n-terminus and employs the irrelevant scFv 14B7 as a carrier protein^39^. Mutagenic primers were used to alter or eliminate the n-terminal DYKD and to introduce the full FLAG peptide and the n-and c-terminal locations. Briefly, the parental plasmid was methylated using CpG Methyltransferase (M.sssI, NEB) for 1.5 h at 37 °C and then used as template in PCR-based site-directed mutagenesis. The PCR reaction followed the commercial instructions, after which *Dpn*I was added to digest methylated template plasmid. The products were transformed into *E. coli* strain XL1-Blue and sequenced at the University of Texas at Austin Core Facility. The previously described MBP-KEE^40^ ligand was used to detect binding by the parental α-EE antibody. Production and purification of all peptide ligands was performed as previously described^39, 40^.

### Phage production and ELISA

Individual colonies were screened using monoclonal phage ELISA as described previously^20^ to assess scFv expression level and ability to bind the FLAG peptide and variants. High-binding 96-well plates (Costar) were coated with carrier protein presenting various peptide ligands or control protein lacking peptide (4 μg/ml) or anti-c-myc antibody (Sigma 9E10, 2 μg/ml). Purified phage were added to blocked wells and detected with anti-M13-HRP (1:3000 in 2.5% non-fat milk in PBST). Specific peptide binding activity was calculated the ratio of signal on peptide-coated wells to anti-c-myc coated wells = A_450_ (n-DYKD)/A_450_ (anti-c-myc). Secondary screens were performed with phage purified by double precipitation with NaCl-PEG and quantified by plaque formation, as described^41^. ELISAs were repeated with phage serially diluted at known phage concentrations (measured as phage forming units) to assess binding to the panel of peptide variants.

### Fab protein purification, CD and ELISA

For expression as soluble Fabs, the V_H_ or V_L_ domain was amplified and sub-cloned into pFabF vector using conventional cloning or the Gibson assembly method, followed by DNA sequencing. These were expressed and purified as described^41^, with the exception that 10% glycerol was added to all buffers and a 125 mM imidazole wash step was added to selectively elute free light chain.

Circular dichroism experiments were conducted using a Jasco J-815 CD Spectrometer equipped with Brinkman RC20 water bath and Jasco MPTC-490S/15 temperature control cell. Two spectra from independent purifications of EEF15.4 were measured in a 0.1 cm cuvette with 20 μM EEf-15.4 in TBS (20 mM Tris-HCl, 150 mM NaCl, pH 8.4), one sample with and the other without 10% glycerol. For thermal melts, spectra were acquired from 200 to 300 nm between 4 to 90 °C with a ramp rate of 1 °C/minute, data pitch of 2 °C and 1 nm bandwidth. Ten spectra were measured at each temperature with a 200 nm/min scan rate. Origin Pro 8.5 was used to plot spectra analyze melt data (CD minimum as function of temperature) using the Boltzmann sigmoid equation.

To measure Fab activity and specificity, ELISA was performed with high-binding 96-well plates (Costar^TM^) were coated with the naked control carrier protein, n-DYKD and peptide variants (10 μg/mL). After blocking, purified Fabs were serially diluted and detected with goat-anti-human kappa-HRP (1:1000, Southern Biotech, 20060-05). Commercial monoclonal anti-Flag M2-HRP (Sigma-Aldrich, A8592) was used as control antibody. GraphPad Prism 6 was used for representation of all ELISA data and all ELISAs were repeated at least twice with separate ligand, Fab and phage preparations.

### Data availability

The data that support the findings of this study are available from the corresponding author upon reasonable request. OptCDR computer codes are freely available with registration on-line at http://www.maranasgroup.com/submission/OptCDR_2.htm.

## Electronic supplementary material

Supplementary information

Supplementary Datasets 1, 2 & 3
