# Supplementary material for: De novo design of antibody complementarity determining regions binding a FLAG tetra-peptide
Source: Sci Rep. 2017 Aug 31;7:10295. doi: 10.1038/s41598-017-10737-9 (PMC5579192; doi:10.1038/s41598-017-10737-9)
Supplement: Supplementary file 1 — Supplementary information [file 41598_2017_10737_MOESM1_ESM.pdf]

Supplementary data for:

***De novo* design of antibody complementarity determining regions binding a FLAG tetra-peptide**

Kevin C. Entzminger, Jeong-min Hyun, Robert J. Pantazes, Athena C. Patterson-Orazem, Ahlam N. Qerqez, Zach P. Frye, Randall A. Hughes, Andrew D. Ellington, Raquel L. Lieberman, Costas D. Maranas, Jennifer A. Maynard

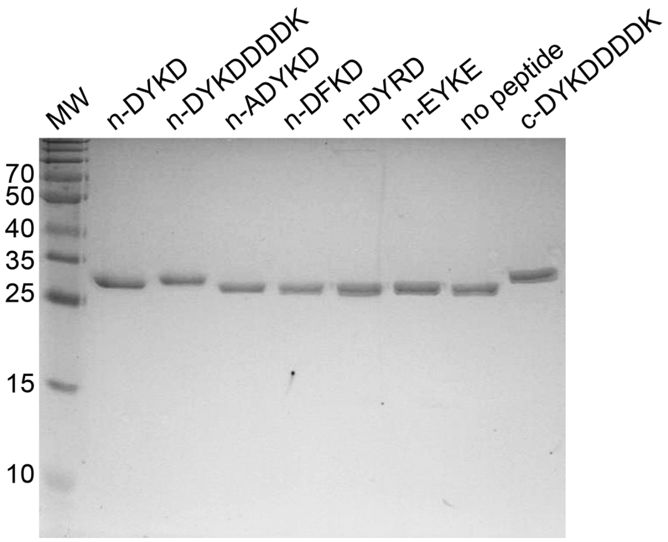

Figure S1. Peptide ligands expressed as fusions to a carrier protein. The 14B7 carrier protein (28 kDa) was expressed with FLAG peptides and variants at either the c- or n-terminus. Tagged proteins were expressed as soluble protein in the *E. coli* periplasm, prior to purification by osmotic shock followed by immobilized metal affinity chromatography and size exclusion chromatography with a Superdex 75 column. Protein purity and size was assessed by SDS-PAGE; molecular weight markers (kDa) are indicated.

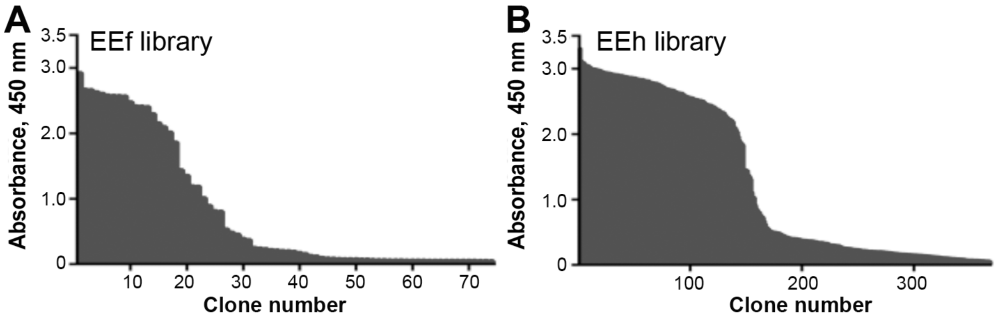

Figure S2. Expression levels of designed antibody library as phage-displayed scFv. ELISA was used to assess the relative levels of full-length scFv expression on phage among (a) the EEf library and (b) the EEh library members. Phage were grown in a 96-well plates and 50 ul transferred to ELISA wells coated with anti-c-myc antibody, followed by detection with anti-M13-HRP antibody. The raw absorbance at 450 nm is reported for each clone tested and reflects the phage production level as well as scFv incorporation, both of which are affected by scFv expression.

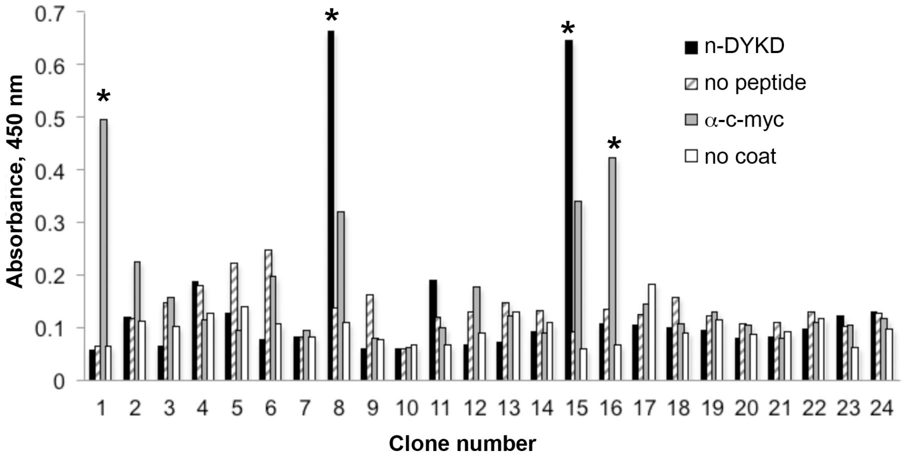

Figure S3. Secondary screen to select scFv clones for further analysis. For a secondary screen of the EEf library, phage ELISA was used to assess scFv expression via binding to the anti-c-myc antibody and peptide-specific binding via binding to DYKD sequence fused to the n-terminus of a carrier protein. Controls included naked carrier protein and uncoated wells. Shown are the raw absorbance values from single wells during screening, which are expected to be in the linear dose-response or background ranges of the assay. Four clones exhibited high level expression based on anti-c-myc binding activities (marked with *), two of which also exhibited strong peptide-binding activities and were selected for further analysis.

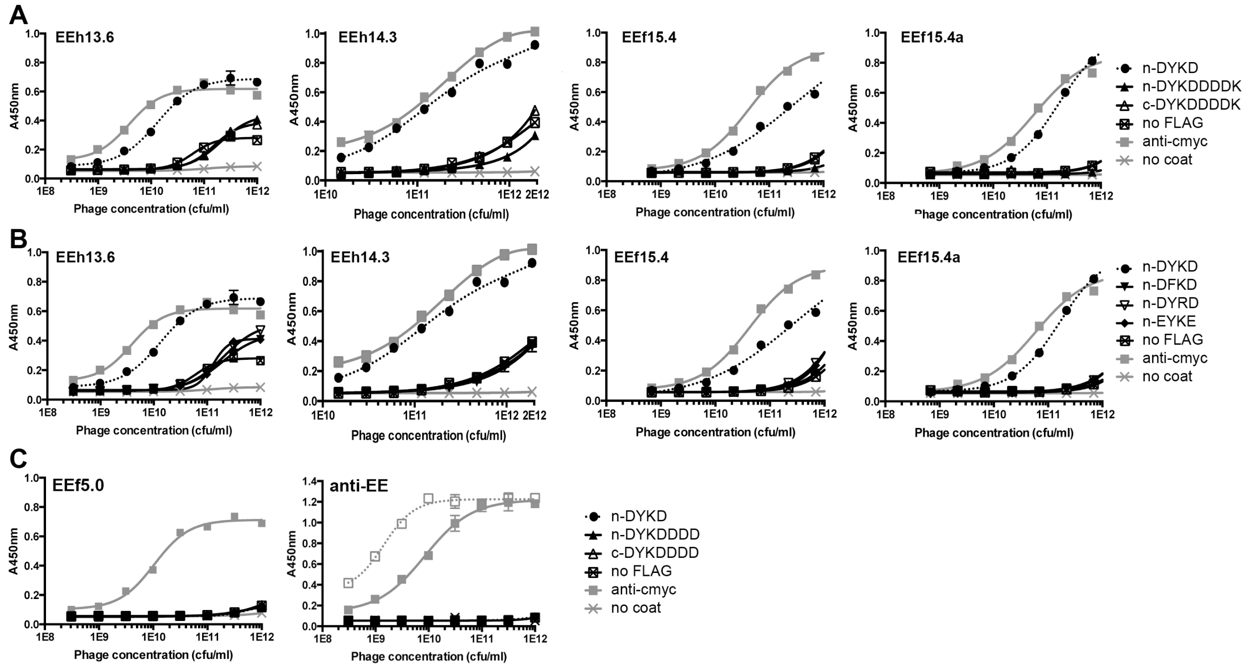

Figure S4. Full ELISA plots of scFv-phage screening. Purified phage preparations were titrated on wells coated with various peptide ligands presented at the n- or c-terminal position of the same carrier peptide. Controls included the anti-c-myc antibody to assess scFv expression levels, the EE peptide to assess recognition of the peptide bound by the scaffold antibody and a no coat control to assess non-specific binding. (a) To evaluate the role of peptide placement, phage displaying scFvs were assessed for peptide binding to carrier protein with no tags, with an n-terminal DYKD, n-terminal DYKDDDDK and c-terminal DYKDDDDK. (b) To assess the specificity of peptide recognition, phage were assessed for binding to variants of the DYKD peptide, presented at the n-terminus: n-DFKD, n-DYRD and n-EYKE. The ELISAs were repeated at least twice using independently purified peptide carrier proteins and independent phage preparations. (c) Control scFvs include EEh5.0, a well-expressed but non-FLAG-binding design, and the anti-EE scFv that served as the framework donor, that does not exhibit binding to any FLAG tag. Shown is the average signal and range for data collected from at least two replicate experiments.

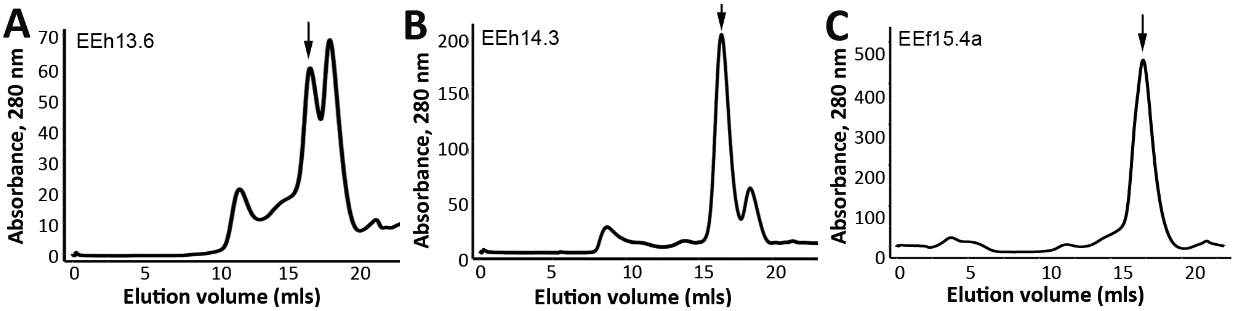

Figure S5. *De novo* designed Fab antibodies express at high yield. The Fabs were each expressed as soluble periplasmic proteins in 250 ml bacterial cultures, purified by osmotic shock followed by immobilized metal affinity chromatography and size exclusion chromatography. Shown are the preparative traces on a Superdex S200 column for (a) Fab EEh13.6, (b) Fab EEh14.3 and (c) Fab EEf 15.4a. The monomeric Fab peak is indicated with an arrow; monomeric fractions were collected for further analysis by CD and ELISA.

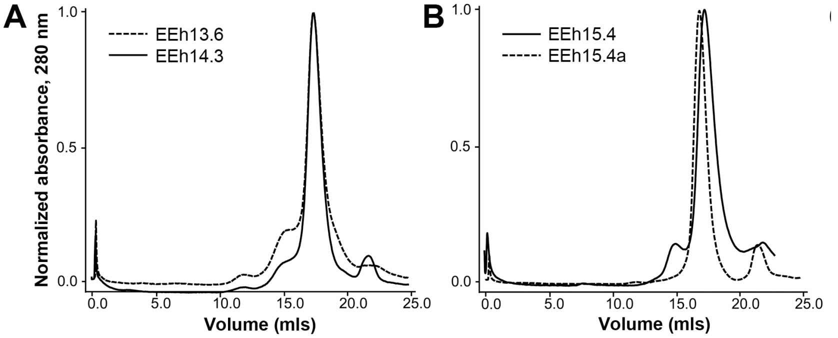

Figure S6. Analytical SEC of purified Fab proteins after two months of storage. Fab proteins were purified via hexa-histidine tag and preparative size exclusion chromatography, as shown in the main text. To assess protein stability, the proteins were subjected to analytical size exclusion chromatography after two months of storage at 4 °C. (a) Fabs EEh13,6 and EEh 14.3; (b) EEh15.4 and EEh15.4a. Shown is the normalized absorbance at 280 nm from a Superdex S200 column.

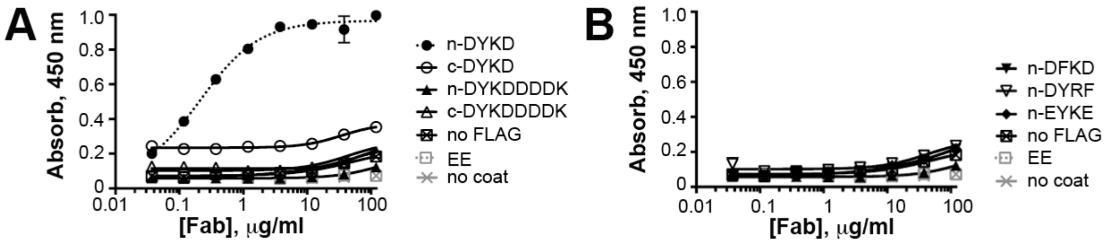

Figure S7. Binding specificity of the EEh15.4a Fab. The purified 15.4a Fab protein was assessed for binding to different FLAG and control peptides presented by the same carrier protein using an ELISA. (a) Binding was assessed for core and full-length FLAG tags at the n- or c-terminal position. (b) Binding was assessed for conservative changes to the n-DYKD ligand: DFKD, DYRD or EYKE and used in ELISAs. The experiment was repeated at least twice with different protein preparations; shown is the average signal and standard deviation from at least two replicate experiments.

Table S1. Alignment of CDR sequences for EEf library models chosen for screening. CDRs from the acceptor framework scFv are also shown. For model derivatives, only amino acid changes from the parent model are shown. CDRs are numbered using Kabat standards, and CDRs are defined according to OptCDR's sequence-based rules.

	V_L CDRs																																	
	CDR1																CDR2							CDR3										
scFv name	24	25	26	27	27A	27B	27C	27D	27E	28	29	30	31	32	33	34	50	51	52	53	54	55	56	89	90	91	92	93	94	95	95A	95B	96	97
Acceptor	R	S	S	Q	S	I	V	H	S	N	G	N	T	Y	L	E	K	V	S	N	R	F	S	F	Q	G	S	L	V	P	-	-	P	T
EEf5.0	R	S	S	Q	-	-	-	-	-	G	S	R	A	N	L	E	A	G	D	N	R	F	S	S	G	F	E	N	S	Q	A	R	S	M
EEf5.1																																		
EEf15.0	R	S	S	N	-	-	-	-	-	A	R	S	G	S	L	E	D	G	N	N	R	F	S	S	S	W	R	N	Q	G	A	K	S	I
EEf15.2				G							T	K	S	Q																				
EEf15.3																																		
EEf15.4																									A	F	D	Q	T	N	K	Y	V	G
EEf15.5																																		
EEf24.0	R	S	S	S	R	N	V	-	-	G	A	N	D	S	L	E	R	G	N	N	R	F	S	G	S	Y	D	L	S	M	K	-	Q	A
EEf24.2																																		
EEf24.5																																		

	V_H CDRs																																					
	CDR1												CDR2											CDR1														
scFv name	26	27	28	29	30	31	32	33	34	35	35A	35B	50	51	52	52A	52B	53	54	55	56	57	58	93	94	95	96	97	98	99	100	100A	100B	100C	100D	100E	101	102
Acceptor	G	Y	S	L	S	T	S	G	M	G	V	N	H	I	Y	-	-	W	D	D	D	K	R	A	R	R	G	G	S	S	H	Y	Y	A	M	-	D	Y
EEf5.0	G	D	I	G	S	S	Y	T	V	N	-	-	H	S	S	G	G	Q	E	A	R	T	R	A	H	S	R	A	M	Q	L	F	D	S	G	G	K	E
EEf5.1		Y					H																															
EEf15.0	G	F	S	I	K	G	A	N	V	N	-	-	H	V	R	-	-	G	D	A	S	T	R	A	D	R	K	M	Y	S	F	Y	S	G	G	-	E	A
EEf15.2																																						
EEf15.3															N			D	S	G	E	A																
EEf15.4																																						
EEf15.5																									K		D	G	W	N	Y	F	Q		A		D	I
EEf24.0	A	F	S	M	G	T	S	G	V	N	-	-	H	I	N	T	-	S	S	E	G	K	R	A	L	D	A	I	M	K	S	G	G	-	-	-	R	W
EEf24.2																										K	S	M	A	R	N						D	
EEf24.5	G	W			A		Q																															

Table S2. Alignment of CDR sequences for EEh library models chosen for screening. Prepared as Table S1.

	V_H CDRs																																					
	CDR1												CDR2												CDR3													
scFv name	26	27	28	29	30	31	32	33	34	35	35A	35B	50	51	52	52A	52B	52C	53	54	55	56	57	58	93	94	95	96	97	98	99	100	100A	100B	100C	100D	101	102
**Acceptor**	G	Y	S	L	S	T	S	G	M	G	V	N	H	I	Y	-	-	-	W	D	D	D	K	R	A	R	R	G	G	S	S	H	Y	Y	A	M	D	Y
**EEh1.0**	G	Y	S	I	T	R	S	S	A	G	V	N	H	N	G	A	-	-	S	Q	D	S	K	R	A	M	F	D	Q	V	G	R	G	-	-	-	H	W
**EEh1.1**																										G		K	N		M							Y
**EEh1.2**														I					D	R	S		T															
**EEh1.4**		D		L		S	Y	N																														
**EEh1.5**															S				D	K	Y	G	S															
**EEh2.0**	G	D	S	V	T	S	Y	N	A	G	V	N	H	T	A	S	-	-	G	E	S	N	K	R	S	R	K	N	M	A	-	-	-	-	-	-	G	W
**EEh2.2**														V	G				R	S	E		Q															
**EEh2.3**																										K	T	M	R									
**EEh2.4**																									V	K	N	S	R	G							A	F
**EEh3.0**	G	R	S	Y	T	A	Q	G	V	N	-	-	H	S	R	D	N	S	E	G	G	A	T	R	S	A	G	-	-	-	-	-	-	-	-	-	D	F
**EEh3.1**																									G	M	A											
**EEh3.3**		A	T		K	S																																
**EEh4.0**	G	G	S	L	T	R	S	N	M	A	V	N	H	A	N	G	K	-	T	M	G	E	S	R	A	G	W	D	N	Y	Y	E	G	-	-	-	R	F
**EEh5.0**	G	E	S	A	S	V	N	G	V	N	-	-	H	S	G	D	K	G	N	S	H	A	T	R	A	G	Y	R	N	Y	W	K	G	-	-	-	E	F
**EEh5.2**		D		V		I	T																															
**EEh5.5**																S	Q				D		K															
**EEh5.6**	D	F	N	G	Q	A	T																															
**EEh6.0**	G	D	S	L	S	T	S	A	M	G	V	N	H	G	S	G	K	-	T	V	A	E	S	R	A	K	S	-	-	-	-	-	-	-	-	-	G	F
**EEh6.1**		F		V				K																														
**EEh6.2**						S	F	N	A																													
**EEh6.3**																F	G		N	K	V	D	Q															
**EEh6.5**																									S	R	G										M	Y
**EEh7.0**	G	D	S	L	T	S	Y	S	A	G	V	N	H	M	R	-	-	-	T	S	G	E	A	R	V	A	F	K	S	I	M	D	G	-	-	-	G	W
**EEh7.2**														V					S	E		N																
**EEh7.3**					S		T	A	M																													
**EEh7.4**																									D	G	W		Q	M	Y	E					A	F
**EEh8.0**	G	V	S	Y	E	N	S	G	V	N	-	-	H	S	G	S	Q	T	H	K	D	A	T	R	S	M	K	R	G	-	-	-	-	-	-	-	A	V
**EEh8.1**		E		M	S	G	Q	A																														
**EEh8.3**																D		A	N		G	E																
**EEh8.4**																									A	S											L	
**EEh8.5**		E	T		N	A																																
**EEh9.0**	G	Y	T	F	S	S	G	D	V	N	-	-	H	S	K	D	Q	G	E	N	G	A	T	R	A	R	F	K	G	S	N	-	-	-	-	-	D	Y
**EEh9.1**																									V	T	R		E		G						A	F
**EEh11.0**	G	D	S	V	T	S	F	N	A	G	V	N	H	M	S	S	-	-	N	G	D	K	Q	R	D	G	F	Y	Q	A	M	K	G	-	-	-	R	Y
**EEh13.0**	G	D	S	V	T	S	Y	S	A	G	V	N	H	G	A	-	-	-	-	V	M	S	T	R	A	K	S	T	G	R	Y	-	-	-	-	-	D	F
**EEh13.4**														L	G					A			V															
**EEh13.6**				L	S		F	N																														
**EEh14.0**	G	D	S	L	S	S	Y	N	A	G	V	N	H	V	G	-	-	-	-	A	M	S	T	R	V	R	N	E	W	S	G	-	-	-	-	-	A	F
**EEh14.3**														M	A				G	V																		
**EEh14.4**														G	A					L		N	I															

Table S3. EEf model characteristics.

scFv name	Interaction energy (kcal/mol)	Contacts[a] (V_H)	Polar contacts[b] (V_H)	Peptide burial[c] (Å)
**EEf5.0**	-609	60 (34)	12 (7)	16.7 ± 1.9
**EEf15.0**	-191	78 (37)	8 (3)	13.3 ± 1.8
**EEf24.0**	-452	52 (43)	10 (6)	18.1 ± 2.3

[a]Defined as the number of atoms predicted to be within 3 Å of the FLAG peptide. Contacts contributed by the heavy chain are shown in parentheses.
[b]Identified with PyMOL. Contacts contributed by the heavy chain are shown in parentheses.
[c]Measured as the distance of all peptide atoms to a fixed plane within the scFv core.

Table S4. EEh model characteristics. Prepared as Table S3.

scFv name	Interaction energy (kcal/mol)	Contacts (V_H)	Polar contacts (V_H)	Peptide burial (Å)
**EEh1.0**	-335	35 (21)	2 (2)	16.1 ± 1.6
**EEh2.0**	-339	29 (9)	3 (2)	18.9 ± 2.3
**EEh3.0**	-392	37 (24)	7 (4)	14.2 ± 1.7
**EEh4.0**	-206	21 (12)	3 (2)	17.3 ± 2.5
**EEh5.0**	-476	59 (26)	4 (1)	14.3 ± 1.9
**EEh6.0**	-351	21 (12)	1 (0)	17.9 ± 2.2
**EEh7.0**	-297	25 (12)	7 (3)	15.2 ± 2.5
**EEh8.0**	-298	30 (20)	6 (4)	17.5 ± 1.6
**EEh9.0**	-129	60 (9)	5 (0)	15.0 ± 2.1
**EEh11.0**	-226	34 (20)	5 (4)	17.3 ± 2.3
**EEh13.0**	-326	35 (14)	10 (5)	17.7 ± 1.7
**EEh14.0**	-328	48 (23)	6 (4)	16.0 ± 1.9
